# Lévy noise improves the electrical activity in a neuron under electromagnetic radiation

**DOI:** 10.1371/journal.pone.0174330

**Published:** 2017-03-30

**Authors:** Juan Wu, Yong Xu, Jun Ma

**Affiliations:** 1 Department of Applied Mathematics, Northwestern Polytechnical University, Xi’an, China; 2 School of Mathematics & Information Science, Beifang University of Nationalities, Yinchuan, China; 3 Potsdam Institute for Climate Impact Research, Potsdam, Germany; 4 Department of Physics, Humboldt University Berlin, Berlin, Germany; 5 Department of Physics, Lanzhou University of Technology, Lanzhou, China; State University of New York, UNITED STATES

## Abstract

As the fluctuations of the internal bioelectricity of nervous system is various and complex, the external electromagnetic radiation induced by magnet flux on membrane can be described by the non-Gaussian type distribution of Lévy noise. Thus, the electrical activities in an improved Hindmarsh-Rose model excited by the external electromagnetic radiation of Lévy noise are investigated and some interesting modes of the electrical activities are exhibited. The external electromagnetic radiation of Lévy noise leads to the mode transition of the electrical activities and spatial phase, such as from the rest state to the firing state, from the spiking state to the spiking state with more spikes, and from the spiking state to the bursting state. Then the time points of the firing state versus Lévy noise intensity are depicted. The increasing of Lévy noise intensity heightens the neuron firing. Also the stationary probability distribution functions of the membrane potential of the neuron induced by the external electromagnetic radiation of Lévy noise with different intensity, stability index and skewness papremeters are analyzed. Moreover, through the positive largest Lyapunov exponent, the parameter regions of chaotic electrical mode of the neuron induced by the external electromagnetic radiation of Lévy noise distribution are detected.

## Introduction

The neuronal system is extremely complex and contains a large number of neurons, where the signals are transferred through electrical activity and extensive signal informations are processed. The electrical activities show various modes with appropriate external forcing that are made up of different neuron states such as resting, spiking, bursting and even chaotic states. The electrical spike-burst activities of neuron are usually measured by a recurrent transition between resting state and firing state[1]. The different electrical activity modes of a neuron and their abundant dynamical behaviors have been researched extensively. The typical neuron models such as FitzHugh-Nagumo (FHN) model, Hodgkin-Huxley (HH) model, Hindmarsh-Rose (HR) model etc. have been investigated theoretically and numerically[2–6]. The dimensionless three-variables HR neuron model, simplified from the original HH neuron model, mainly describes the neuronal activities of spiking-bursting behavior of membrane potential in a single neuron. According to the bifurcation analysis, when adjusting the model parameters, the mode transition of electrical activities appears observably. In addition, the dynamical behaviors of bifurcation, phase transition, chaos and synchronization etc. in electrical activities were investigated largely [2, 7, 8]. Actually, the electrical activity of neuronal system is rather complex and many factors should also be considered, such as the effect of ion channels etc. Based on the Faraday’s law of induction, the fluctuation or change of potentials of neuron can generate magnet field in the external environment, and the electricalal activities of neuron will be modulated under the feedback effect of magnet field. Furtherly, the fluctuation of membrane potentials of neuron could influence the distribution of the inner or external electromagnetic field. Therefore the magnetic flux across membrane and electromagnetic induction effect are considered, and then the general HR model in an isolate neuron has been extended to an improved model of four variables with magnetic flux [9–11].

The external electromagnet radiation can induce the magnet flux on membranes. And the neuron usually shows evident spiking frequency adaptation when it is adjusted by cellular electrical fields [12]. Clinical effects of the transcranial electrical stimulation with weak currents are remarkable when the electrical fields of low amplitude act on the ten billions of brain neuron. Gamma oscillations induced by carbachol in rat hippocampal slices have an internal rate-limiting dynamic and time precision that govern the systematic susceptibility to low-frequency weak electrical fields[13]. Also, CA1 pyramidal neurons affected by DC fields show the characteristic polarization, whose mechanism underlying the profiles is investigated by applying optical imaging and patch-clamp recordings[14]. Those external forcing induced by external radiation-induced magnet flux of electrical fields usually can be described as random noises. Originally, it was believed that noises often have a negative effect on neurons, such as destroy equilibrium state, submerge valuable signal etc. However, with the development of noise research, its positive effect has been widely discovered, e.g. applying noises to enhance the characteristic performance in some fields [15–21]. In the researches, the effect of the external magnet field is described by Gaussian while type of noise which is just an idealized noise for simplicity. Actually the neuron environment is complex and the induced random noises are usually of non-Gaussian distribution. Lévy noise is a kind of non-Gaussian noise with stochastic jumps and heavy tail, which can greatly describe the complex biological and neuronal environment [22, 23]. Some researcheres have done a large number of interesting works about Lévy noise. Nurzaman et al. extend Lévy walks model of one of the simplest creature Escherichia coli, based on biological fluctuation framework[24]. Dubkov et al. examine the steady-state time characteristics of anomalous diffusion in the form of Lévy flights in a symmetric bistable quartic potential[25]. Xu et al. investigate stochastic resonance phenomenon in a FitzHugh-Nagumo system induced by an additive Lévy noise [24, 26]. Xu et al. also study the state switch of protein concentration in the gene transcriptional regulatory system [27]. Those researches focus on the nonlinear behaviors in some usual systems induced Lévy noise. However, the researches in an improved HR model with electromagnetic radiation have not been investigated. Therefore, it is valuable to study the effect of non-Gaussian Lévy noise on the neuron of improved HR model under magnetic flow.

In this work, the firing patterns of the improved dimensionless four-variables HR model excited by non-Gaussian Lévy noise and some dynamical behaviors are investigated. In Sect.2, the brief introduction of the improved HR model under the magnetic flux across the membrane of neuron and non-Gaussian Lévy noise is given. Then, the numerical simulation results and discussions of the electrical activities, the spatial phase, the time points of neural firing, the stationary probability distribution functions of the membrane potential of the neuron and chaotic electrical mode are present in Sect. 3. Finally, the concluding remarks are provided in Sect. 4.

## Model

The HR model of neuronal activity mainly focuses on the spiking-bursting behavior of the membrane potential in a single neuron and produces main dynamical properties in electrical activities. The electromagnetic induction exists in the fluctuation of membrane potentials of the neuron and can induce the magnet flux on the membrane potentials. Thus the effect of the electromagnetic induction is considered and the improved mathematical HR model is described as follows [9, 10]:

$$\left\{ \begin{array}{l} \dot{x}=y-ax^{3}+bx^{2}-z+I_{ext}-k_{1}W(\varphi )x\\ \dot{y}=c-dx^{2}-y\\ \dot{z}=r[s(x+1.6)-z]\\ \dot{\varphi }=kx-k_{2}\varphi +\varphi_{ext}, \end{array}\right.$$ (1)

where *x*,*y*,*z* denote the membrane potential, slow current associated with recovery variable and adaption current, respectively. *φ* describes the magnetic flux across the membrane of neuron. *W*(*φ*) respects the memory conductance of a magnetic flux-controlled memristor and is used to describe the coupling between magnetic flux and membrane potential of neuron here, which is shown as the following

$$W(\varphi )=\frac{dq(\varphi )}{d\varphi }=\lambda +3\mu \varphi^{2}.$$ (2)

The physical significance of *W*(*φ*)*x* can be understood through the following equation

$$\frac{dq(\varphi )}{dt}=\frac{dq(\varphi )}{d\varphi }\frac{d\varphi }{dt}=W(\varphi )V=k_{1}W(\varphi )x,$$ (3)

where the variable *V* is the induced electromotive force. *k*_1_*W*(*φ*)*x* respects the feedback current on membrane potential when magnet flux is changed. *k*_1_ is the feedback gain. The terms of *kx* and *k*_2_*φ* denote the membrane potential-induced changes on magnet flux and leakage of magnet flux respectively. *I*_*ext*_ is the external forcing current and we consider the periodical type *I*_*ext*_ = *A*cos *wt*. *φ*_*ext*_ is external field or electromagnet radiation-induced magnet flux on the membrane. According to the Maxwell electromagnetic induction theorem, the electrical activity of neuron can be changed due to the effect of internal bioelectricity of nervous system (e.g. fluctuation of ion concentration inside and outside of cell). The effect of external field or electromagnet radiation-induced magnet flux on the membrane *φ*_*ext*_ is described by random Lévy noise *ζ*(*t*), as the intricate externanl environment induced by the firing and impulse activities of neurons. On the base of the *α*-stable distributions theory and fractional order derivatives, Lévy noise *ζ*(*t*) is the form derivative of α-stable Lévy process *L*(*t*) and the explicit formula of the characteristic function of *L*(*t*) can be scanned to the refs [18, 22, 23, 28].

**Fig 1:**
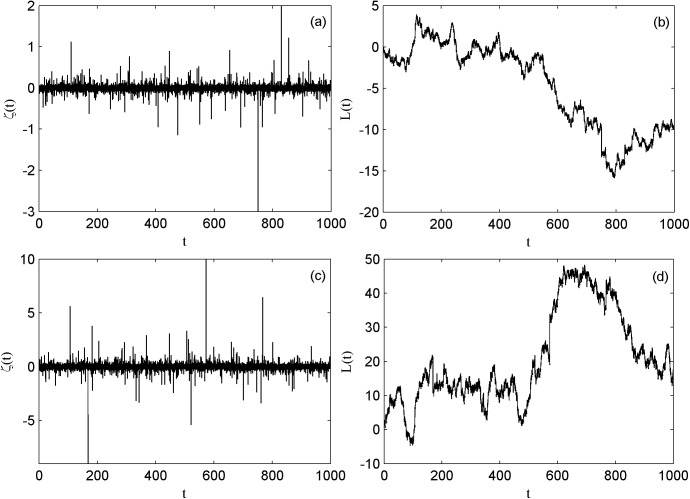
the sequences of Lévy noise and Lévy process. (a) Lévy noise of *α* = 1.8, *β* = 0, D = 0.05; (b) Lévy process of *α* = 1.8, *β* = 0, D = 0.05; (c) Lévy noise of *α* = 1.8, *β* = 0, D = 0.5; (d) Lévy process of *α* = 1.8, *β* = 0, D = 0.5.

In the following presentation of Lévy noise *ζ*(*t*) with three parameters *α*, *β*, *σ*. *α*(0 < *α* ≤ 2) is the stability index. The skewness parameter *β*(−1 ≤ *β* ≤ 1) measures the symmetry of Lévy noise. *σ*(*σ* > 0) represents the scale parameter *σ* = *D*^1/*α*^ and *D* is the Lévy noise intensity. The sample paths of Lévy process can be described through Janicki-Weron algorithm[29]. The diagrams of Lévy noise and Lévy process are shown in Fig 1.

## Results

In order to get the numerical series of membrane potential of the HR model, the fourth order Runge-Kutta algorithm is applied to calculate the model. Here, some basic parameters of the model are fixed as *a* = 1.0, *b* = 3.0, *c* = 1.0, *d* = 5.0, *r* = 0.006, *k* = 1.0, *s* = 4.0 and the other parameters of the magnetic flux for *k*_1_, *k*_2_, *λ*, *μ*, *φ*_*ext*_ and *I*_*ext*_ are differently changed.

**Fig 2:**
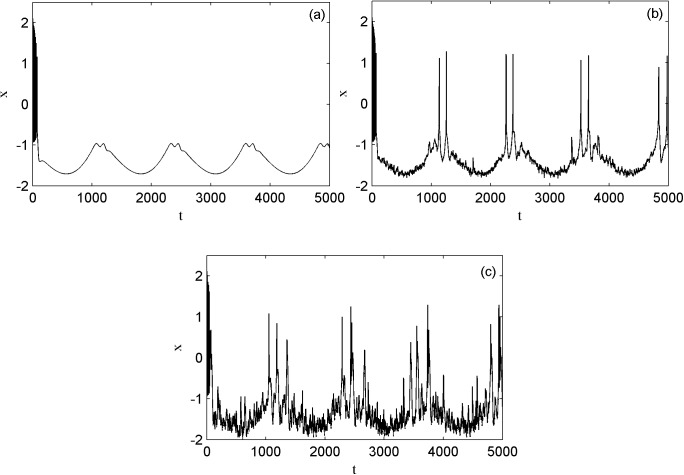
The time series of the membrane potential of neuron under different external radiation-induced magnet flux.

In Fig 2, the electrical activities of the neuron are plotted with and without Lévy noise. The parameters of magnetic flux are *k*_1_ = 1.0, *k*_2_ = 0.5, *λ* = 0.1, *μ* = 0.02, the parameters of *I*_*ext*_ and *φ*_*ext*_ are *A* = 1.6, *ω* = 0.005, *α* = 1.9 and *β* = 0. Then the different activity modes are obtained through tuning the external electromagnet radiation-induced magnet flux of Lévy noise on the membrane. When there is no external electromagnet radiation-induced magnet flux of Lévy noise, the electrical activities of the neuron are quiescent. When the external electromagnet radiation-induced magnet flux of Lévy noise with the intensity 0.1 acts on the membrane, the resting state of the neuron is excited into the spiking state with two spikes. Further, when the Lévy noise intensity is increased to 1.0, the spiking state with two spikes of the electrical activities is excited to have more spikes.

**Fig 3:**
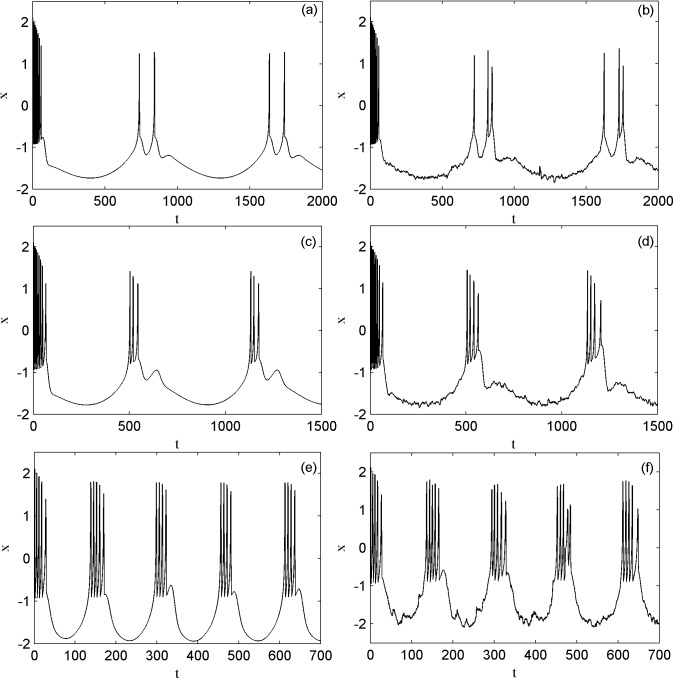
The time series of the membrane potential of neuron under different external radiation-induced magnet flux.

Then we investigate other interesting modes of the electrical activities. Fig 3 shows the different electrical activity models without noise and with the external electromagnet radiation-induced magnet flux of Lévy noise on the membrane, through tuning the external forcing current frequency. The parameters of magnetic flux are *k*_1_ = 1.0, *k*_2_ = 0.5, *λ* = 0.1, *μ* = 0.02, the external forcing current amplitude of *I*_*ext*_ is *A* = 1.6. As the external forcing current frequency is increased from 0.007 to 0.01 and then to 0.04 displayed in the panels of (a), (c) and (e), the number of the spikes in one periodic electrical activity is increased from two to three and then to four respectively. The external forcing current frequency can change the electrical activity modes without noise. In addition, the effect of the external electromagnet radiation-induced magnet flux of Lévy noise on three different modes of electrical activities are shown in the panels of (b), (d) and (f), where the stability index and the skewness parameters of Lévy noise are fixed as *α* = 1.8, *β* = 0 and the intensity is 0.05, 0.06, 0.4 successively. Then compared to the left and right panels, it is found that under the appropriate intensity Lévy noise can induce another new spike in a periodic electrical activity, i.e. Lévy noise improves the firing of electrical activity of neuron.

**Fig 4:**
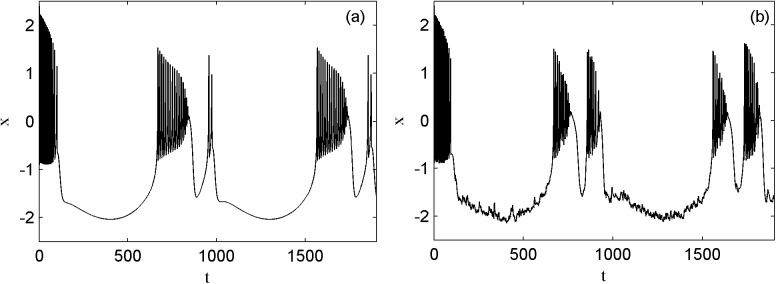
The time series of the membrane potential of neuron under different external radiation-induced magnet flux.

Fig 4 shows another different electrical activity modes of the neuron. The parameters of magnetic flux are *k*_1_ = 0.4, *k*_2_ = 0.5, *λ* = 0.4, *μ* = 0.02, the external forcing current parameters of *I*_*ext*_ are *A* = 3.0 and *ω* = 0.007 in panel (a). The time series of the membrane potential of the neuron induced by Lévy noise with *α* = 1.9, *β* = 0.0, *D* = 1.0 in panel (b). Compared the modes in panels (a) and (b), apparently the external electromagnet radiation-induced magnet flux of Lévy noise promotes the bursting states to degenerate and the spiking states to transform into the bursting states.

**Fig 5:**
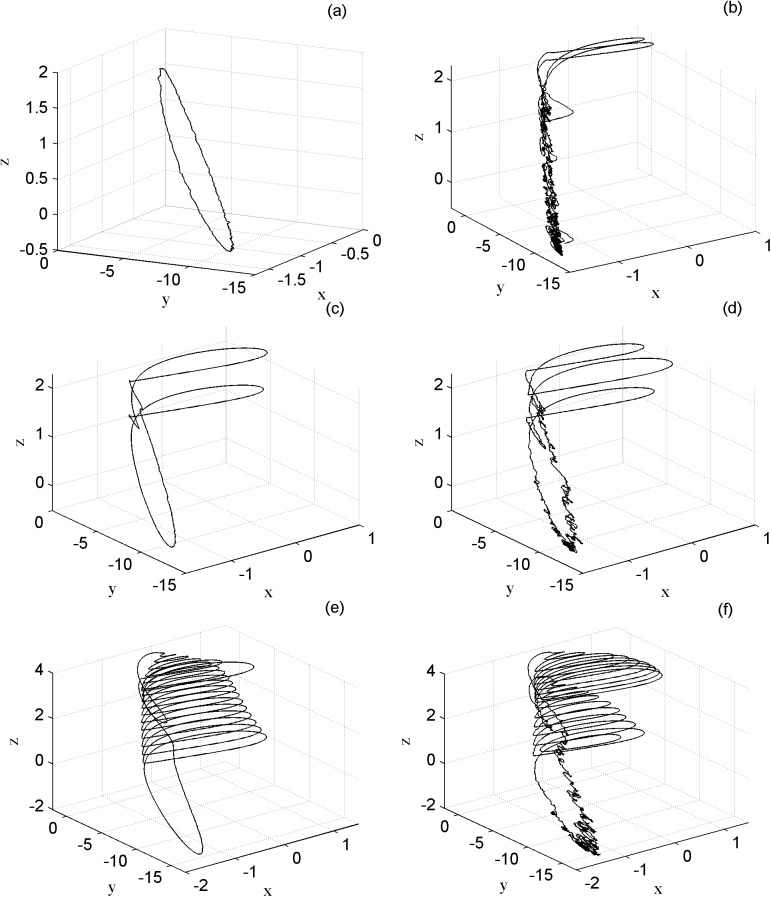
The spatial phase diagrams of electrical activity under different external radiation-induced magnet flux (a), (c) and (e) no noise; (b), (d) and (f) Lévy noise.

Next, the transfer of the spatial phase of a periodic electrical activity is analyzed in order to investigate the effect of Lévy noise on the neuron. Fig 5 shows the spatial phase diagrams of a periodic electrical activity without noise and with the external electromagnet radiation-induced magnet flux of Lévy noise. The parameters of magnetic flux are fixed as *k*_1_ = 1.0, *k*_2_ = 0.5, *λ* = 0.1, *μ* = 0.02, the parameters of *I*_*ext*_ are *A* = 1.6 and *ω* = 0.003. The spatial phase diagram of a periodic electrical activity is shown in panel (a). Also the spatial phase diagram induced by the external electromagnet radiation-induced magnet flux of Lévy noise with the parameters of *α* = 1.9, *β* = 0 and *D* = 0.1 is shown in panel (b). Compared to panels (a) and (b), the appropriate external electromagnet radiation-induced magnet flux of Lévy noise excites the resting state of the electrical activity to be the spiking state. Then the external forcing current frequency is increased to 0.007, the spatial phase diagram is shown in panels(c) and (d) respectively. It is observed that the proper external electromagnet radiation-induced magnet flux induces the greater spikes of the spiking state of the electrical activity. In addition, compared to the parameters of panel (c), *k*_1_ decreases to 0.4, *λ* increases to 0.4, *A* increases to 3.0, and other parameters remain unchanged, the spatial phase diagrams are shown in panel (e). The transfer of the spatial phase diagrams of the electrical activity are shown in panel (f) when the external electromagnet radiation-induced magnet flux of Lévy noise with *α* = 1.8, *β* = 0, *D* = 1.0 acts on the membrane potential. It is shown that the proper Lévy noise causes the different phase transitions from the resting state to the spiking state and to the busting state.

**Fig 6:**
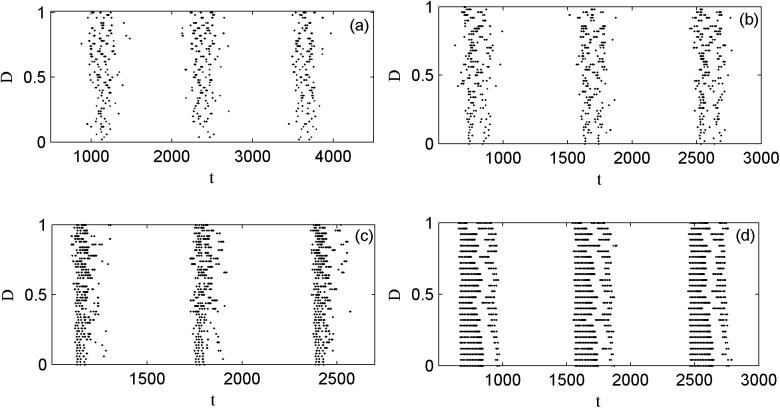
The time points of the neural firing as the Lévy noise intensity.

Further, the time points of the electrical activities with the spiking states and the bursting states are displayed in Fig 6, which mainly shows the neural firing of the electrical activities as the change of noise intensity. The threshold of the neural firing of is setted as 0, when the membrane potential goes over the threshold, the time points of the firing of neuron are marked. In Fig 6(A), the paremeters are *k*_1_ = 1.0, *k*_2_ = 0.5, *λ* = 0.1, *μ* = 0.02, *A* = 1.6, *α* = 1.9, *β* = 0, *ω* = 0.005. When the Lévy noise intensity is 0, there is no marked time point, i.e., no firing of the neuron. As the intensity of Lévy noise exceeds 0 and is increased gradually to 1.0, the time points of the firing of neuron become more and more. As *ω* is increased to 0.007 and other parameters are unchanged, the time points are shown in Fig 6(B). When the neuron is not excited by the external electromagnet radiation-induced magnet flux of Lévy noise, the spiking state of a periodical electrical activity presents two spikes only. Then the Lévy noise intensity is increased to 1.0, the time points of the neural firing become much more. As *ω* is increased to 0.01 once again, the time points are shown in Fig 6(C). A periodical electrical activity without the effect of Lévy noise presents three spikes. When the noise intensity is increased to 1.0, the time points of the neural firing get much more. In addition, the paremeters of Fig 6(D) are adjusted into *k*_1_ = 0.4, *λ* = 0.4, *A* = 3.0 and *ω* = 0.007, the bursting states and the spiking states both exist. When the Lévy noise intensity is increased to 1.0, it is found that the amount of the time points of the spiking states increase and the time points of the bursting states decrease.

**Fig 7:**
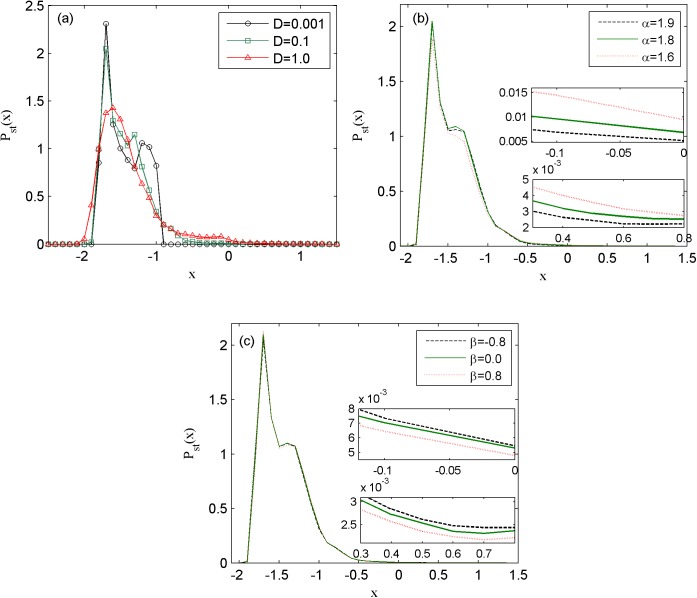
the stationary probability distribution function of membrane potential of neuron under different external radiation-induced magnet flux of Lévy noise.

Moreover, the stationary probability distribution functions of the membrane potential for the different noise intensity, stability index and skewness parameter of Lévy noise are exhibited in Fig 7. The parameters are fixed as *k*_1_ = 1.0, *k*_2_ = 0.5, *λ* = 0.1, *μ* = 0.02, *A* = 1.6, *ω* = 0.005, *D* = 0.1, *α* = 1.9 and *β* = 0. In panel (a), as the Lévy noise intensity is increased from 0 to 0.1 and then to 1.0, the stationary probability distribution functions of the state that is less than -1.0 decreases, while the stationary probability distribution functions of the state that is large than -1.0 increases gradually, and the bimodal stationary probability distribution function changes into unimodal function. It means that increasing Lévy noise intensity can improve the electrical activities of the neuron. In panel (b), the effect of adjusting stability index *α* of Lévy noise on stationary probability distribution function is shown. When *α* is decreased from 1.9 to 1.8 then to 1.6, i.e., the jump amplitude of Lévy noise is augmented and the stability index of Lévy noise is reduced, the general shapes of the stationary probability distribution functions are almost unchanged, as the firing points in the whole electrical activity are extremely less. In fact, the stationary probability of the firing states that is shown in the internal small figures is gradually increased, namely, the decreasing of the stability index of Lévy noise improves the firing of neuron. In panel (c), the effect of altering skewness parameter *β* of Lévy noise on stationary probability distribution function is exhibited. As *β* is increased from 0 to 0.8 and decreased to -0.8, the general shapes of the stationary probability distribution functions are almost unchanged. However, the increasing of *β* lessens the the stationary probability of the firing states and the decreasing of *β* improves the the stationary probability of the firing states shown in the internal small figures, namely, the upward or downward skewing of the jumps of Lévy noise suppresses or improves the firing of neuron.

**Fig 8:**
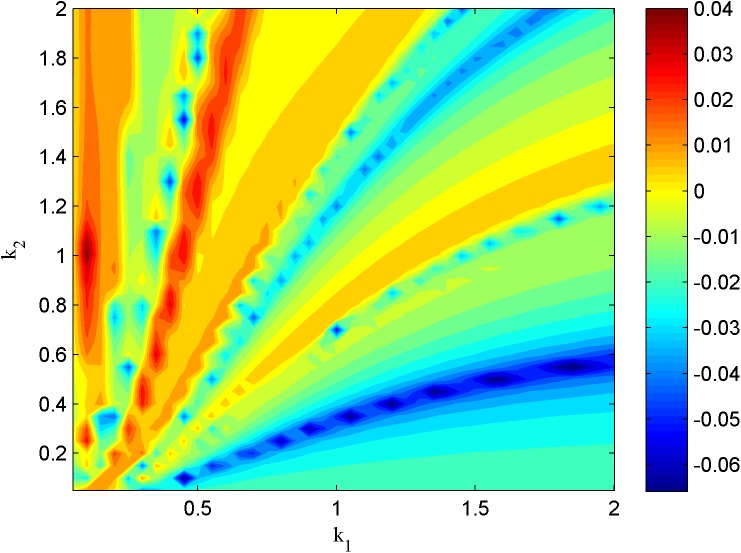
The largest Lyapunov exponent in the two-parameter region (*k*_1_,*k*_2_).

**Fig 9:**
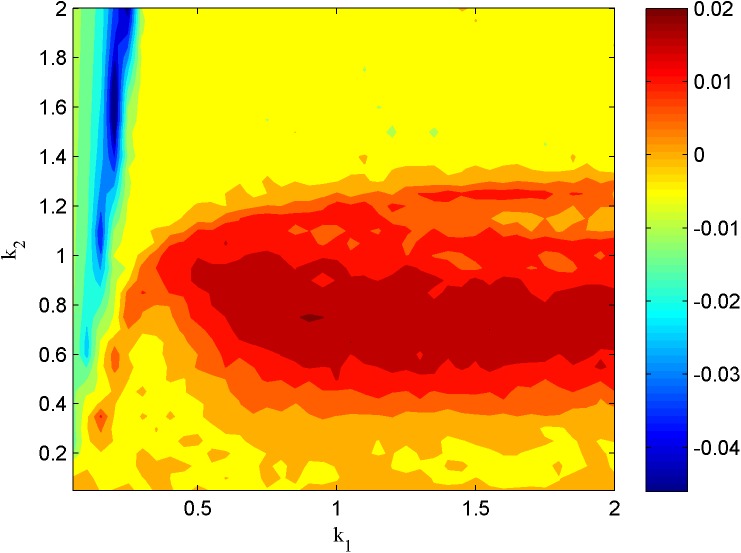
The largest Lyapunov exponent in the two-parameter region (*k*_1_,*k*_2_).

In addition, the chaotic parameter regions could also be detected by calculating the largest Lyapunov exponent spectrum beyond zero, and the parameter regions of the largest Lyapunov exponents are shown in Figs 8 and 9. In Fig 8, the neuron under the external forcing periodical current and the external radiation-induced magnet flux of Lévy noise with *λ* = 0.1, *μ* = 0.02, *A* = 3.0, *ω* = 0.2, *α* = 1.9, *β* = 0 and *D* = 0.001. In Fig 9, the neuron under the external forcing constant current and the external radiation-induced magnet flux of Lévy noise with *λ* = 0.1, *μ* = 0.02, *A* = 3.5, *α* = 1.9, *β* = 0 and *D* = 0.01. In the parameter regions of Figs 8 and 9 where the Lyapunov exponent is positive, the neuron can present chaotic electrical mode. The neuron in the magnetic flow effect can show the appropriate electrical modes with chaotic properties according to the different external forcing and Lévy simulation.

## Concluding remarks

Considering the fluctuations of the internal bioelectricity induced by electromagnetic induction, the dimensionless stochastic HR model under the effect of non-Gaussian Lévy noise is given and the electrical activities of neuron are calculated. It is revealed that the electromagnetic induction of Lévy noise distribution can cause the mode transition of electrical activities and spatial phase diagram. The rest states of the electrical activities of neuron is excited to be firing states with two spikes and three spikes. The spiking states with two, three and four spikes in a periodic electrical activities can be induced to be spiking states with three, four and five spikes or even more spikes. More, the spiking states can be changed into bursting state. Then the time points of the firing states are shown for different parameters, which demonstrate that the increase of Lévy noise intensity can greatly heighten the firing of neuron. Next, the stationary probability distribution functions of membrane potential are calculated. It is found that as Lévy noise intensity is gradually added from 0 to 1.0, the probability distribution of resting states is reduced and the probability distribution of firing states is enhanced obviously. The stability index and the skewness parameters do not influence the general shapes of the stationary probability distribution. However, the decreasing of the stability index of Lévy noise can improves the firing of neuron and the upward or downward skewing of the jumps of Lévy noise suppresses or improves the firing of neuron. Moreover, the neuron under the electromagnetic induction of Lévy noise distribution is on the chaotic electrical mode. In general Lévy noise could improve electrical activity in a neuron under electromagnetic radiation.
